# Retraction: Long noncoding RNA *VPS9D1-AS1* augments the malignant phenotype of non-small cell lung cancer by sponging microRNA-532-3p and thereby enhancing *HMGA2* expression

**DOI:** 10.18632/aging.204829

**Published:** 2023-06-15

**Authors:** Xiao Han, Tianren Huang, Junqing Han

**Affiliations:** 1 Guangxi Medical University, Cancer Hospital, Nanning 530021, China; 2 Cancer Center, Shandong Provincial Hospital Affiliated to Shandong University, Jinan 250021, China

**Keywords:** non-small cell lung cancer, microRNA-532-3p, high mobility group AT-hook 2, *VPS9D1-AS1*

**This article has been retracted:** Aging has completed its investigation of this paper. We found overlap between several transwell assay images used for multiple figures and data published by different authors. Specifically, in Figure 2E, the motility image for A549 si-NC overlaps an image from [1]. In Figure 2F, the invasion image for A549 si-NC overlaps an image from [2]. In Figure 4E, the H460 agomir-NC image is a duplicate of an image from [3]. In Figure 6D, the motility image for A549 si-NC overlaps an image from [4]. In Figure 6E, the invasion image for H460 si-VPS9D1-AS1 + antagomir-523-3p overlaps an image from [5]. In Figure 7F, invasion panels for H460 si-NC and si-VPS9D1-AS1+pc-HMGA2 overlap panels from [6,7]. Finally, panels for A549 agomir-NC in Figure 4D and H460 si-VPS9D1-AS1+pcDNA3.1 in Figure 7F overlap panels from [8]. As a result, all of the listed authors agreed that the article should be retracted.

The Administration of the Shandong University, Guangxi Medical University and their respective affiliated hospitals was notified about the retraction by Aging Journal because of the improper use of multiple pictures included in this manuscript. The authors apologize for any inconvenience this retraction has caused for the scientific community.
